# Neurotransmitter alterations in seasonal affective disorder

**DOI:** 10.1038/s41598-026-37634-4

**Published:** 2026-01-30

**Authors:** B. Spurny-Dworak, G. Dörl, P. Stöhrmann, M. Klöbl, A. Igumnova, M. Rothenberg, J. Donath, P. Handschuh, C. Schmidt, W. Bogner, M. Spies, E. Winkler-Pjrek, D. Winkler, Rupert Lanzenberger

**Affiliations:** 1 Department of Psychiatry and Psychotherapy, Medical University of Vienna, Waehringer Guertel 18-20, 1090 Vienna, Austria; 2 Comprehensive Center for Clinical Neurosciences and Mental Health (C3NMH), Medical University of Vienna, Vienna, Austria; 3 Karl Landsteiner University of Health Sciences, Krems an der Donau, Austria; 4 Department of Biomedical Imaging and Image-Guided Therapy, High Field MR Center, Medical University of Vienna, Vienna, Austria; 5 Christian Doppler Laboratory for MR Imaging Biomarkers (BIOMAK), Medical University of Vienna, Vienna, Austria; 6 Comprehensive Center for AI in Medicine (CAIM), Medical University of Vienna, Vienna, Austria

**Keywords:** Seasonal, Depression, SAD, GABA, Glutamate, Diseases, Neurology, Neuroscience

## Abstract

Seasonal affective disorder (SAD) is a type of unipolar depression characterized by depressive symptoms mainly during the cold season, which were often linked to alterations in the serotonergic system. It is assumed that other neurotransmitter systems, such as glutamate and GABA, are similarly affected. Hence, we investigated differences in glutamate and GABA between SAD patients and healthy control subjects using magnetic resonance spectroscopy imaging (MRSI). Fourteen SAD patients (11 female, 36 ± 11 years) and 14 sex- and age-matched healthy controls, were scanned once between October and February using multi-voxel 3D-GABA-edited MEGA-LASER MRSI at 3 T. Mean GABA+ and Glx (glutamate + glutamine) to total creatine (tCr) ratios were calculated in five brain regions. Mann–Whitney-U-Tests were performed for each region and neurotransmitter ratio independently as well as correlation analyses between neurotransmitter ratios and clinical scores, respectively. A significant reduction in GABA+/tCr ratios in the hippocampus (*p*_corr_ = 0.049) between SAD patients and healthy individuals was revealed. No significant changes in other brain regions or correlations with the investigated clinical scores were shown. Our findings of altered GABA concentrations in the hippocampus are in line with neurotransmitter alterations across other subtypes of depression, hinting towards common neurobiological mechanisms and highlights the interplay between environmental factors and neurotransmitter systems.

**Supplementary Information:**

The online version contains supplementary material available at 10.1038/s41598-026-37634-4.

## Introduction

Seasonal affective disorder (SAD) is a form of major depressive disorder (MDD), characterized by recurrent depressive episodes during fall and winter^1^. Seasonal variations in symptom severity are believed to be linked to light exposure^1^. Therefore, bright light therapy (BLT) is the gold standard for treatment of SAD, showing high success rates^2,3^. However, neurobiological underpinnings of SAD remain speculative.

Similar to MDD, SAD is mainly associated with altered serotonergic neurotransmission. Variations in the serotonin systems along the annual cycle have often been described in positron emission tomography (PET) studies^4^. Seasonal alterations in serotonin transporter (SERT)^5,6^, monoamine oxidase A (MAO-A)^7^ or the serotonin 1A receptor^8^ were reported. Additionally, an impact of BLT on monoamine oxidase A levels in the brain^7^ and SERT binding was shown^9^.

While the serotonergic system plays an important role in the pathophysiology of MDD and SAD, especially the main inhibitory and excitatory neurotransmitter systems of the brain, gamma-amino butyric acid (GABA) and glutamate, show interplay and modulatory effects on serotonin levels. An interesting seasonal link between serotonin and GABA was suggested in a study by Li et al. reporting seasonal rhythms of hippocampal GABA and serotonin concentrations in rats^10^.

Both GABA and glutamate have shown substantial implications in the pathophysiology of MDD^11,12^. Several studies using magnetic resonance spectroscopy (MRS), a non-invasive MR-based method for the in vivo quantification of GABA and glutamate, reported reduced levels of glutamate and/or GABA across different brain regions^13–15^. Hence, it is of interest to further elucidate the implications of GABA and glutamate in MDD and different subtypes, including SAD.

Since the serotonergic system shows seasonal variations also in healthy individuals, we have investigated seasonal alterations in GABA and glutamate levels in a healthy study cohort in our previous study^16^. Our analyses suggested seasonal stability of these neurotransmitter in different subcortical regions (the hippocampus, putamen, pallidum and thalamus) and the insula.

Thus, to further explore changes in the main neurotransmitter system of the brain in SAD, we compared levels of GABA and glutamate across different brain regions between patients suffering from SAD and age- and sex-matched healthy control subjects in the fall or winter season, when symptoms were more pronounced.

## Experimental procedures

### Study design

Fourteen patients suffering from SAD (11 female, mean age ± SD = 36 ± 11 years) and 14 sex- and age-matched (± 2 years) control subjects, were scanned once during fall or winter season (between October and February). This study was approved by the ethical committee of Medical University of Vienna, Austria (EK 1482/2019) and conducted according to the Declaration of Helsinki. Written informed consent was obtained from all study participants.

### Participants

All patients had a DSM-5 diagnosis of a recurrent major depressive disorder with a seasonal pattern and were currently in a depressive episode. They further had a global seasonality score ≥ 10 on the seasonal pattern assessment questionnaire (SPAQ)^17^ and a structured interview guide for the Hamilton rating scale for depression–seasonal affective disorder (SIGH-SAD) score of ≥ 20 at screening visit^18^. Patients were untreated for SAD and free from any other present psychiatric disorder, suicidal ideations, substance abuse or major internal or neurological disease. Moreover, patients with concomitant neuropsychological medication or bright light therapy during the last 6 months were excluded from the study.

Healthy control subjects had no internal, neurological or psychiatric disorders, no history of substance abuse and reported no lifetime use of psychotropic agents or antidepressants.

All subjects were free of contraindications for MRI and were not pregnant or currently breastfeeding. Urine drug and pregnancy tests (for women) were performed prior to each MRI session.

### Psychological tests

The SPAQ and the SIGH-SAD were conducted by a trained psychiatrist at the day of the MR measurement.

### MRS measurements and data analysis

**Fig. 1:**
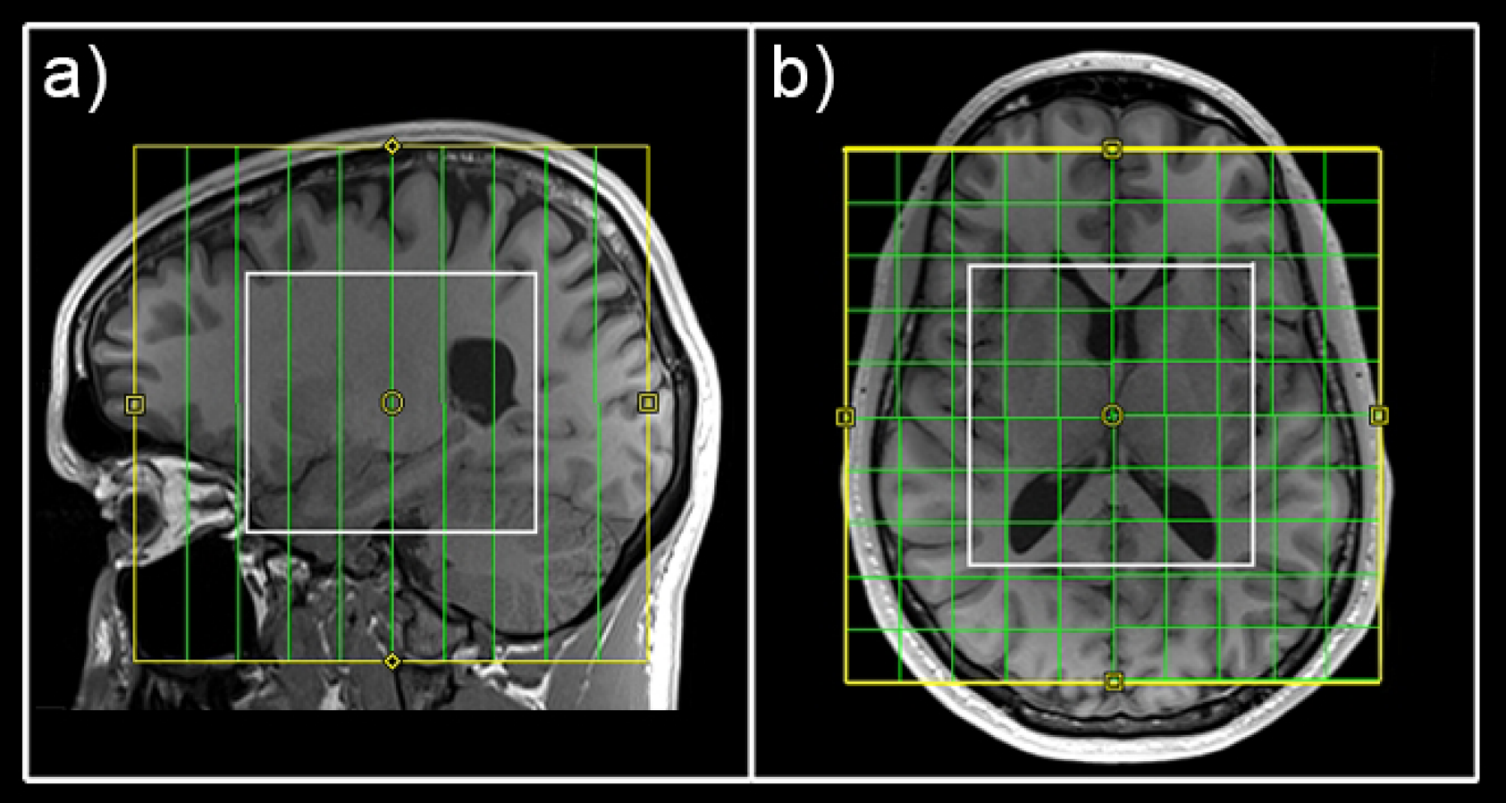
Placement of the field of view (yellow) and volume of interest (white) to cover all regions of interest.

MRI measurements were conducted on a 3 Tesla Prisma MR system (Siemens Medical, Erlangen, Germany) at the High-field MR Center, Department of Biomedical Imaging and Image-guided Therapy, Medical University of Vienna with a 64-channel head coil. Structural T1-weighted images (TE = 1800 ms, TR = 2.37 ms, 208 slices, 288 × 288 matrix size, voxel size 1.15 × 1.15 × 0.85 mm) were acquired prior to each MRSI scan for accurate volume of interest (VOI) placement and region-of-interest (ROI)-based quantification of MRS data. For MRS acquisition a 3D GABA-edited MEGA-LASER MRSI sequence with real-time correction for rigid body motion and center frequency changes^19^ described in Bogner^20^ was used (TR = 1600 ms, TE = 68 ms, volume of interest (VOI) = 80 (l-r) × 90 (a-p) × 80 (s-i) mm^3^, field of view (FOV) = 160 × 160 × 160 mm^3^, 32 acquisition weighted averages, two-step phase cycling in a total scan time of 15:09 min). The acquired matrix size of 10 × 10 × 10 (i.e., approximately 4 cm^3^ nominal voxel size) was interpolated to a 16 × 16 × 16 matrix (i.e., approximately 1 cm^3^ nominal voxel size) during spectral processing steps. During the EDIT‐ON acquisition, MEGA‐editing pulses (60 Hz Gaussian pulses of 14.8 ms duration) were set to 1.9 ppm, editing the coupled 4CH_2_ triplet of GABA resonating at 3.02 ppm^21–23^. VOI selection via LASER and low‐power and wide‐bandwidth GOIA pulses enabled MEGA editing with an echo time of 68 ms^20^. For real‐time correction, volumetric, dual‐contrast, echo planar imaging-based navigators that update center frequency and head‐position changes for each pair of EDIT‐ON/OFF acquisitions were used (i.e. with a repetition time of 1.6 s, updated every 3.2 s). Advanced Siemens shimming procedure with manual adjustments was applied. The VOI was placed parallel to the anterior commissure–posterior commissure line to cover the hippocampus and insula bilaterally (see Fig. 1).

### MRSI data analysis

**Fig. 2:**
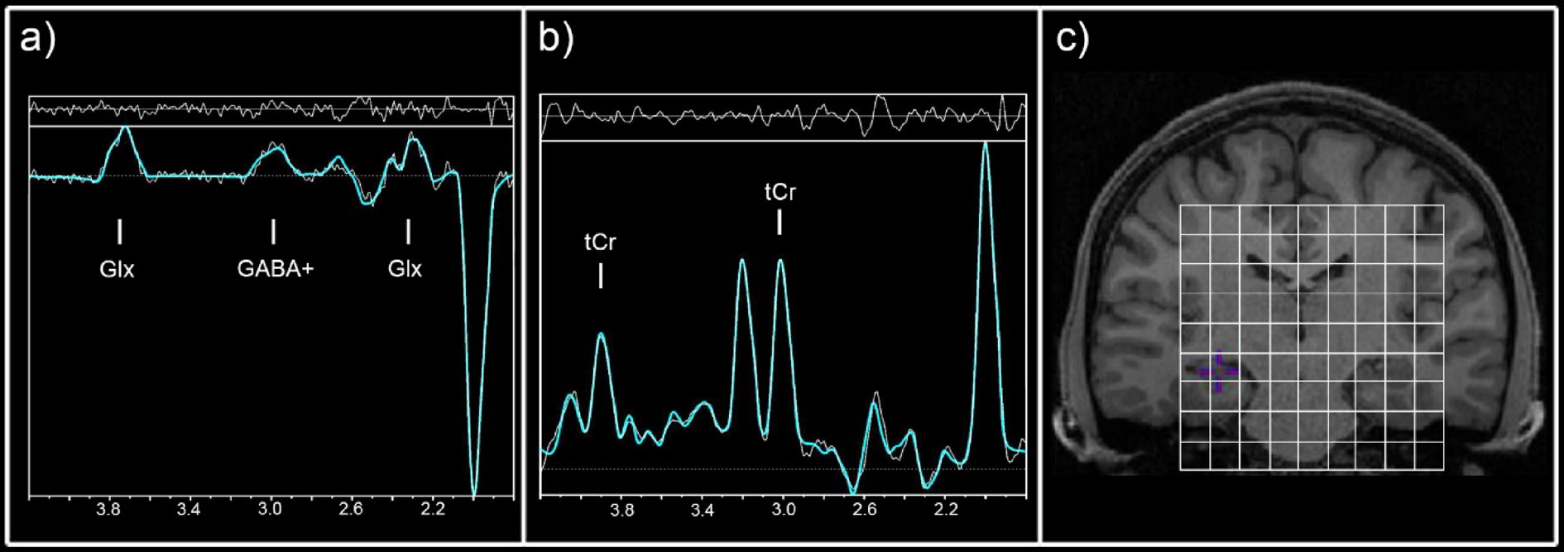
Exemplary difference (**a**) and unedited spectra (**b**) of the hippocampus at the distinct voxel position in the volume of interest depicted in (**c**). Concentrations of GABA+ (GABA+ macromolecules) and Glx (glutamate + glutamine) were extracted from the difference spectrum (**a**), whereas concentrations of tCr (total creatine) were derived from the unedited spectra (**b**).

For MRSI data analysis a combination of MATLAB (R2013a, MathWorks, Natick, MA, USA), Bash (4.2.25, Free Software Foundation, Boston, MA, USA), MINC (2.0, MINC Tools, McConnell Brain Imaging Center, Montreal, QC, Canada) and LCModel software (6.3–1, S. Provencher, LCModel, Oakville, ON, Canada) was used. Two different basis sets, one for the non-edited spectra (containing tCr among others) and one for the difference spectrum (including GABA+ and Glx)^24^ were created with the GAMMA library. Cramér–Rao lower bounds (CRLB) thresholds were set at 30% for the quantification of all spectra within the VOI and maps and spectra were visually inspected. An ROI-based quantification, described in Spurny et. al ^25^, was used for the analysis of GABA+ and Glx ratios to total creatine (GABA+/tCr and Glx/tCr) in the hippocampus, insula, putamen, pallidum and thalamus. In short, masks of each ROI were derived from the automated segmentation of structural images using FreeSurfer. Maps of GABA+, Glx and tCr were interpolated to the resolution of structural images (288 × 288 × 208) and overlaid with the derived masks. Mean ratios of GABA+/tCr and Glx/tCr, across all voxels within each region, were calculated. ROIs with < 90% valid voxels, due to CRLB thresholds or insufficient voxel coverage, were excluded from subsequent analyses. Exemplary spectra are depicted in Fig. 2 and Supplementary Fig. S1.

### Statistical analyses

Statistical analyses were conducted using SPSS Statistics (v24.0, 2010, SPSS, Inc., an IBM Company, Chicago, United States of America). Mann–Whitney-U-Tests were used for the comparison of GABA+/tCr and Glx/tCr ration within each ROI between patients suffering from SAD and healthy controls, respectively. Sidak correction was used for correction of multiple comparison.

Moreover, to examine the relationship between symptom severity and neurotransmitter levels in SAD patients, Kendall rank correlation coefficients were calculated between neurotransmitter ratios (GABA+/tCr and Glx/tCr) within each ROI and SPAQ or SIGH-SAD scores, respectively. Again, Sidak correction was used to correct for multiple comparison. For quality measures, CRLB, full width at half maximum (FWHM) values and signal-to-noise ratios (SNR) within each ROI were compared between groups using Mann–Whitney-U-tests.

## Results

**Fig. 3:**
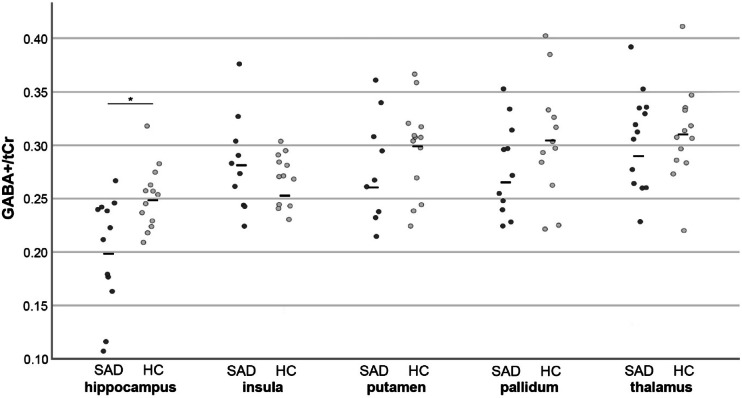
Individual GABA+/tCr ratios within each brain of SAD patients (dark grey) and sex- and age-matched healthy control subjects (light grey). Horizontal lines represent mean ratios within each group. GABA+ = GABA+ macromolecules, tCr = total creatine, SAD = seasonal affective disorder.

SAD patients showed mean ± SD SIGH-SAD scores of 25 ± 4 and SPAQ scores of 14 ± 3. Median GABA+/tCr and Glx/tCr ratios of each group and ROI are represented in Supplementary Table S1. Mann–Whitney-U-tests revealed significant differences GABA+/tCr ratios in the hippocampus (*p*_corr._ = 0.049), see Fig. 3. No significant differences were shown in other ROIs or in Glx/tCr ratios.

Correlation analyses revealed no significant correlations between GABA+/tCr or Glx/tCr ratios and SIGH-SAD or SPAQ scores, respectively. SNR and CRLB values revealed slightly poorer spectral quality and fits in the patient cohort (see Supplementary Table S2 and Supplementary Table S3). No differences were shown in FWHM between groups.

## Discussion

Our analyses revealed significant reduced hippocampal GABA+/tCr ratios in SAD patients compared to age- and sex-matched healthy individuals. No changes were shown in Glx/tCr ratios or other brain regions. Moreover, no correlations between SIGH-SAD or SPAQ scores with neurotransmitter ratios remained significant after correction for multiple comparison.

Although seasonal affective disorder is closely linked to dysfunctions in the serotonergic system^6–8,26^, the main inhibitory neurotransmitter system tends to play an important role in its pathophysiology. Several studies revealed altered concentrations of GABA and glutamate in depressed patients across different brain regions^13–15,27^. Here, we reveal for the first time similar reductions in GABA concentrations in SAD.

Significant reductions of GABA content were found in the hippocampus. The hippocampus, as part of the limbic system, is a key region involved in the pathophysiology of depression^28,29^. Beside changes in neurotransmitter content, altered connectivity was frequently reported^27,30,31^. In our previous works we could identify the hippocampus as an important target for neurotransmitter adaptions after antidepressant treatment^32,33^, hormone therapy^34^ or neuroplastic events^35^. Reductions in GABA or glutamate content of the hippocampus, as shown in our study, are often associated with poorer episodic memory, impaired neuroplasticity or deficits in working memory^36–38^. Hence, a variety of symptoms in SAD or MDD may be attributed to this brain region.

In the context of SAD, seasonality and light exposure are key components of pathophysiological processes and are thought to be closely linked to altered serotonin metabolism^8,39^. Studies investigating seasonality of hippocampal neurotransmitter concentrations and function showed varying results. In rats, seasonal rhythms of hippocampal GABA and serotonin concentrations^10^ and photoperiod effects of the pineal gland on the hippocampus were shown^40^. However, in humans, we could provide evidence for stable concentrations of GABA and glutamate in healthy individuals throughout the year across different brain regions, including the hippocampus^16^. Nevertheless, our results arise from cross-sectional data. Hence future approaches need to clarify longitudinal seasonal changes in GABA and glutamate concentrations in SAD patients.

Interestingly our results reveal similar neurobiological alterations of SAD compared to MDD. While MDD and SAD have distinct symptoms in common (depressed mood, fatigue, changes in sleep and eating behavior, among others), underlying pathophysiological mechanisms potentially differ, which is reflected in different treatment approaches and efficacies. Selective serotonin reuptake inhibitors (SSRIs) are the first-line treatment in MDD, showing success rates of approximately 50%^41,42^. Thereby, the serotonergic system is directly targeted. Nevertheless, we could provide evidence of downstream effects on hippocampal glutamate levels after SSRI intake^33^, highlighting the tight interplay between neurotransmitter systems, especially in the hippocampus. However, SAD is predominantly treated with bright light therapy (BLT) with an effect size above 0.8^2^. On the other hand, BLT used in non-seasonal depression shows tremendously reduced success rates^43^. Thus, these two distinct subtypes of depression show similar neurotransmitter adaptions, but need different treatment approaches. Hence, it can be speculated that neurotransmitter changes, especially in the hippocampus, are symptom specific adaptions but are not underlying pathophysiological effects for the development of SAD. However, it has to be considered, that symptom severity did not correlate with neurotransmitter ratios in our study sample. At this state, reasons remain speculative. Symptom severity can be perceived very individually. Moreover, overall depression scores are potentially related to brain networks and cannot be attributed to single brain regions. However, due to the sample size, an analysis, linking single symptoms to each brain region would have been underpowered in this work, but should be considered in future approaches.

Finally, SAD patients showed higher variations in hippocampal GABA concentrations than healthy individuals, which hints towards different neurobiological subtypes of SAD. However, subtype specific evaluations need bigger sample sizes in future approaches, ideally investigating potential correlations between symptom severity and neurotransmitter changes in a longitudinal setting.

However, this study is not without limitations. The derived GABA spectra, using MRSI at 3 T, include macromolecular contamination affecting the specificity of GABA results. Hence it should be considered that GABA+ results are shown. Moreover, potential movement artifacts lead to partially worse spectral fits in the patient cohort. Nevertheless, all derived maps were visually checked during stringent quality control steps and only spectra passing CRLB thresholds and visual inspection were used for further analyses. Finally, the sample size may be too small to reveal a relationship of neurotransmitter ratios and symptom severity in our study cohort.

## Conclusion

Here we report altered GABA concentrations in the hippocampus, a key region in depression as a part of the limbic system. Thus, in animal studies, the interplay between serotonin and GABA could be shown, when hippocampal concentrations of serotonin and GABA followed seasonal rhythms. While we previously showed seasonal stability of GABA and glutamate concentrations in healthy subjects, significant differences in hippocampal GABA levels in SAD patients are of utter importance for a better understanding of SAD. Moreover, similar patterns of neurotransmitter alterations across different subtypes of depression may hint towards common mechanisms for symptom development and should be considered for precise treatment approaches. Hence, our results highlight the complex interplay between environmental factors and neurotransmitter systems in SAD.

## Supplementary Information

Below is the link to the electronic supplementary material.

Supplementary Material 1

## Data Availability

Due to data protection laws processed data is available from the authors upon reasonable request. Please contact rupert.lanzenberger@meduniwien.ac.at with any questions or requests.
