# Supplementary material for: Neurotransmitter alterations in seasonal affective disorder
Source: Sci Rep. 2026 Jan 30;16:6683. doi: 10.1038/s41598-026-37634-4 (PMC12913925; doi:10.1038/s41598-026-37634-4)
Supplement: Supplementary file 1 — Supplementary Material 1 [file 41598_2026_37634_MOESM1_ESM.pdf]

Supplementary material

Supplementary Table S1: Median neurotransmitter ratios of SAD patients and healthy control subjects within each region of interest. Numbers in brackets represent the 25th and 75th percentiles respectively. GABA+ = GABA + macromolecules, Glx = glutamate + glutamine, tCr = total creatine, SAD = seasonal affective disorder, HC = healthy controls

	GABA+/tCr		Glx/tCr	
	SAD	HC	SAD	HC
**hippocampus**	0.21 (0.16 / 0.24)	0.24 (0.23 / 0.26)	1.58 (1.46 / 1.63)	1.61 (1.36 / 1.73)
**insula**	0.26 (0.24 / 0.31)	0.27 (0.25 / 0.27)	1.57 (1.31 / 1.72)	1.52 (1.45 / 1.62)
**putamen**	0.25 (0.23 / 0.30)	0.31 (0.28 / 0.32)	1.56 (1.42 / 1.78)	1.50 (1.39 / 1.60)
**pallidum**	0.27 (0.23 / 0.31)	0.30 (0.28 / 0.32)	1.72 (1.62 / 2.02)	1.40 (1.37 / 1.56)
**thalamus**	0.30 (0.26 / 0.33)	0.31 (0.30 / 0.33)	1.50 (1.33 / 1.67)	1.39 (1.32 / 1.50)

Supplementary Table S2: Median Cramér Rao Lower Bounds (CRLB) derived from GABA+ and Glx spectral fits of SAD patients and healthy control subjects within each region of interest. Numbers in brackets represent the 25th and 75th percentiles respectively. Moreover, calculated p-values of group differences are depicted. GABA+ = GABA + macromolecules, Glx = glutamate + glutamine, SAD = seasonal affective disorder, HC = healthy controls

	GABA+			Glx		
	CRLB SAD	CRLB HC	p-value	CRLB SAD	CRLB HC	p-value
**hippocampus**	20.0 (17.3 / 21.7)	17.0 (15.7 / 18.0)	0.04	11.4 (9.0 / 12.6)	11.6 (7.4 / 12.5)	0.6
**insula**	16.4 (13.1 / 18.6)	15.1 (14.2 / 17.3)	0.8	10.5 (9.1 / 12.2)	8.6 (8.2 / 9.7)	0.04
**putamen**	14.3 (11.6 / 19.9)	12.2 (10.7 / 13.1)	0.2	10.8 (8.9 / 12.5)	8.1 (6.9 / 9.0)	0.007
**pallidum**	14.9 (11.9 / 19.0)	12.1 (11.1 / 12.8)	0.05	11.1 (9.0 / 12.9)	8.0 (6.8 / 9.4)	0.003
**thalamus**	13.4 (11.3 / 16.1)	11.9 (11.4 / 12.1)	0.15	9.2 (7.8 / 10.1)	7.6 (6.8 / 8.5)	0.09

Supplementary Table S3: Median full width at half maximum (FWHM) and signal-to-noise ratios (SNR) of SAD patients and healthy control subjects within each region of interest. Numbers in brackets represent the 25th and 75th percentiles respectively. Moreover, calculated p-values of group differences are depicted. SAD = seasonal affective disorder, HC = healthy controls

	FWHM			SNR		
	SAD	HC	p-value	SAD	HC	p-value
**hippocampus**	0.072 (0.068 / 0.078)	0.067 (0.062 / 0.084)	0.72	10.3 (9.4 / 15.5)	15.1 (11.6 / 19.6)	0.08
**insula**	0.091 (0.084 / 0.095)	0.089 (0.077 / 0.097)	0.57	11.7 (10.8 / 13.2)	14.7 (12.3 / 16.6)	0.06
**putamen**	0.104 (0.097 / 0.114)	0.101 (0.081 / 0.111)	0.63	12.9 (11.3 / 17.7)	17.9 (15.6 / 19.4)	0.04
**pallidum**	0.102 (0.096 / 0.107)	0.094 (0.078 / 0.107)	0.67	14.1 (10.4 / 15.8)	18.5 (16.0 / 20.6)	0.02
**thalamus**	0.077 (0.065 / 0.084)	0.068 (0.059 / 0.075)	0.07	19.5 (16.9 / 22.2)	22.9 (20.7 / 24.5)	0.02

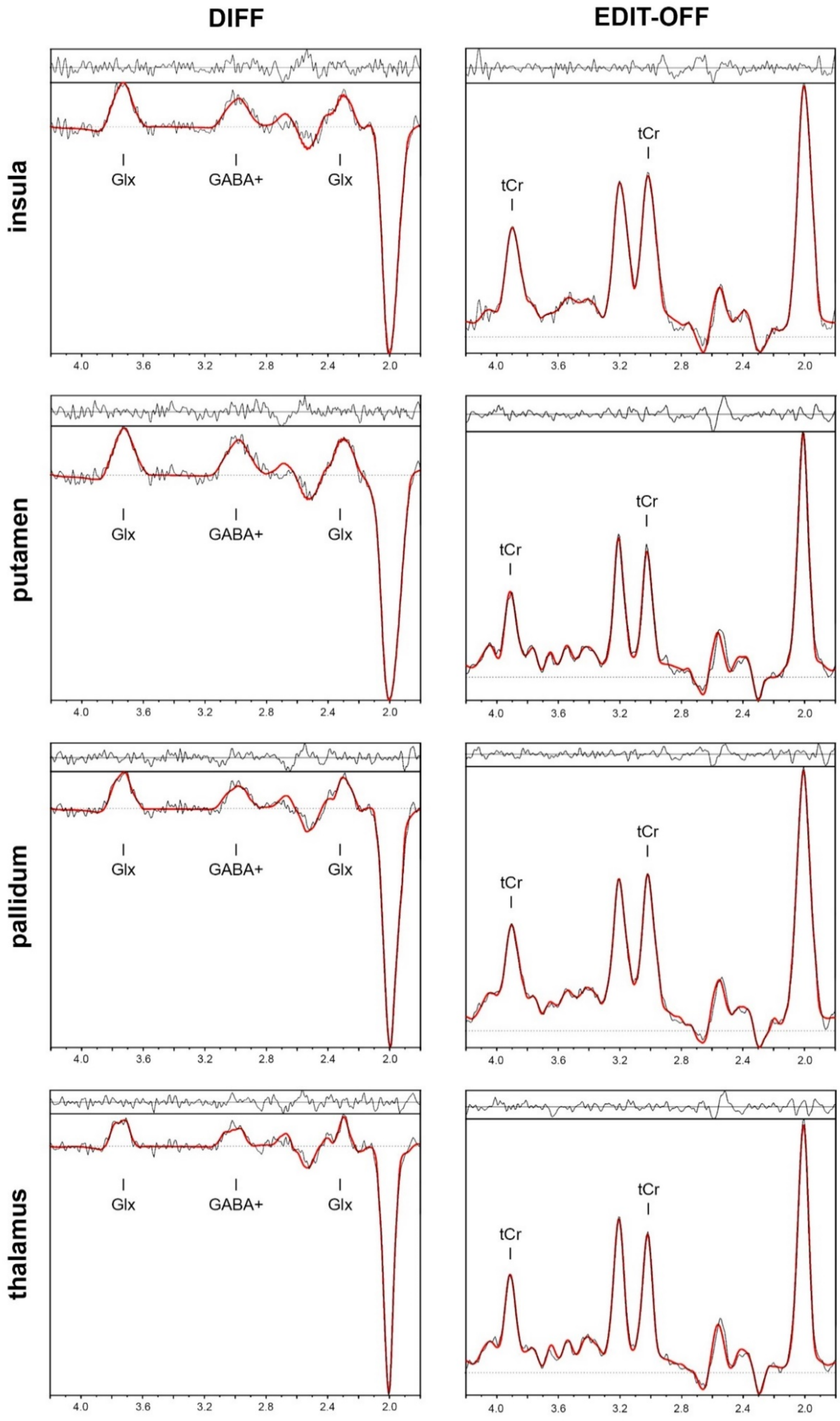

Supplementary Figure S1: Representative difference (DIFF) and unedited (EDIT-OFF) spectra of the insula, putamen, pallidum and thalamus. GABA+ = GABA + macromolecules, Glx = glutamate + glutamine, tCr = total creatine
